# Cancer and the Microbiome of the Human Body

**DOI:** 10.3390/nu16162790

**Published:** 2024-08-21

**Authors:** Lourdes Herrera-Quintana, Héctor Vázquez-Lorente, Maria Lopez-Garzon, Adrián Cortés-Martín, Julio Plaza-Diaz

**Affiliations:** 1 Department of Physiology, Schools of Pharmacy and Medicine, University of Granada, 18071 Granada, Spain; lourdesherrera@ugr.es (L.H.-Q.); hectorvazquez@ugr.es (H.V.-L.); 2 Biomedical Research Center, Health Sciences Technology Park, University of Granada, 18016 Granada, Spain; 3 Biomedical Group (BIO277), Department of Physical Therapy, Health Sciences Faculty, University of Granada, 18171 Granada, Spain; maloga@ugr.es; 4 PROmoting FITness and Health through Physical Activity Research Group (PROFITH), Sport and Health University Research Institute (iMUDS), University of Granada, 18016 Granada, Spain; acortesmartin@ugr.es; 5 APC Microbiome Ireland, School of Microbiology, University College Cork, T12 YT20 Cork, Ireland; 6 Department of Biochemistry and Molecular Biology II, School of Pharmacy, University of Granada, Campus de Cartuja s/n, 18071 Granada, Spain; 7 Instituto de Investigación Biosanitaria IBS.GRANADA, Complejo Hospitalario Universitario de Granada, 18014 Granada, Spain; 8 Children’s Hospital of Eastern Ontario Research Institute, Ottawa, ON K1H 8L1, Canada

**Keywords:** microbiome, cancer, health, diagnostic methods, novel treatments

## Abstract

Cancer remains a public health concern worldwide, with its incidence increasing worldwide and expected to continue growing during the next decades. The microbiome has emerged as a central factor in human health and disease, demonstrating an intricate relationship between the microbiome and cancer. Although some microbiomes present within local tissues have been shown to restrict cancer development, mainly by interacting with cancer cells or the host immune system, some microorganisms are harmful to human health and risk factors for cancer development. This review summarizes the recent evidence concerning the microbiome and some of the most common cancer types (i.e., lung, head and neck, breast, gastric, colorectal, prostate, and cervix cancers), providing a general overview of future clinical approaches and perspectives.

## 1. Introduction

The human microbiome includes all microbes on and inside the human body, and it is closely associated with different dimensions of individual health [1]. The number of bacteria in the body has been estimated to be of the same order as the number of living cells, with a total mass around 0.2 kg [2]. During the past two decades, the interest and understanding of the human microbiome’s role in host physiology has grown considerably. This interest has evolved from descriptive research into mechanistic exploration of causality and molecular insights [3].

In this regard, the gut microbiome regulates host metabolism and physiology, participating in digestion and nutrient availability, and modulating immune responses. Likewise, microbiome composition is influenced by genetic and environmental factors, including diet, which is considered a key regulator of the intestinal microbiome [4]. For instance, a low-fat and high-fiber diet has been seen to decrease markers of inflammation [5]. Additionally, there is growing evidence showing the influence of the microbiome in different related processes, such as its implication in the number and function of neutrophils in visceral adipose tissue. This may vary depending on diet and the presence of obesity [6]. Hence, the microbiome has developed as a central factor in human disease and health, with emerging literature indicating significant implications for conditions like type 2 diabetes or cardiovascular diseases [7]. 

In the context of cancer, the intricate relationships with the human microbiome (also known as oncobiosis) have been recognized as key contributors to the development of the disease as well as modulators of the treatment efficacy. The oncobiome may influence the cancer process through direct interactions with cancer cells, alterations in the tumor microenvironment (TME), or modulation of the immune response [8]. However, a substantial literature gap in terms of the relationships between microbes and cancer still persists, with a few microbes known to directly increase the risk of developing cancer, several known to promote host antitumor immunity, and many more with an undetermined role [9]. Thus, this review summarizes the present evidence concerning the microbiome and some of the most common cancer types (i.e., lung, head and neck, breast, gastric, colorectal, prostate, and cervix cancers), as well as providing a general overview of future clinical approaches and perspectives.

## 2. Cancer Background: Prevalence and Physio-Pathological Overview 

The latest estimates from the International Agency for Research on Cancer (IARC), an agency of the World Health Organization (WHO), highlight the increase in cancer incidence in 2022, with 20 million new cases and 9.7 million deaths [10]. This report, which covers 185 countries and 36 cancer types, estimates that one in five individuals will develop cancer in their lifetime. The number of cancer cases in 2050 will be over 35 million (an increase of 77%) [10]. Lung, breast, and colorectal cancers are the three major cancer types, while the top ten major types, collectively, comprise around two-thirds of cancer cases and deaths globally (this information is collected and summarized in Table 1) [11]. The increased cancer incidence has social and economic consequences. For instance, in the European Union, cancer costs increased from EUR 79 to EUR 86 billion during 2005–2014 [12]. Thus, it is imperative to embrace novel approaches to cancer prevention, an objective which has remained largely unfulfilled for more than a quarter of a century [13].

Cancer initiation is the result of two main cellular events: genetic or epigenetic changes in some genes implicated in suppression/oncogenic signaling pathways, and the subsequent formation of malignant clones [14]. The accumulation of cellular and DNA damage, due to endogenous or exogenous factors, is considered the most significant cause of cancer [15]. On the other hand, cancer processes are linked to inflammation. Chronic inflammation modifies the microenvironment and functions of surrounding cells, which suggests several inflammation pathways that may be regulating cancer promotion [15]. In this line, tumor cells may develop adaptive immune resistance that vigorously switches off an effective antitumor response by the immune system [16]. Furthermore, tumor cells have shown resistance to oxidative damage through increased antioxidant enzyme activity and the intracellular pool of nicotinamide adenine dinucleotide phosphate (NADPH) and glutathione (GSH) [17]. Similarly, other functions, such as autophagy, appear to exert opposing roles depending on the stage: they may help prevent cancer at early stages but support the maintenance and adaptation of established and metastasizing tumors [18].

Prognosis and survival are strongly associated with the stage of the disease, with cancer acquiring a malignant and invasive phenotype at later stages [17]. Cancer metastasis—the principal cancer lethality cause—consists of the cancer cells’ dissemination from a primary lesion to distal organs through several mechanisms, such as escaping immune surveillance or modulating the TME [19]. Cancer metastasis is an inefficient process, with most cancer cells released from the primary tumor dying before creating a distant metastatic tumor, where the pro-inflammatory TME within the extracellular matrix (ECM) present an important role in modulating several processes (e.g., invasion, metastasis, or angiogenesis) [14]. TME modulation is conducted by enrolling and reprogramming non-cancerous cells, remodeling the ECM, and promoting vasculature formation. In this regard, the ECM has a dynamic role in TME creation, influencing cell signaling and tissue biomechanics [20]. During the tumor cells’ dissemination from the primary site to other tissues, the expression of cytoskeletal proteins (e.g., actin, myosin, or intermediate filaments) can be related to the degree of malignancy [21]. Furthermore, abnormal expression of cadherins—a large family of cell surface proteins that participate in adhesion at intercellular adherens junctions—and the destabilization of the cadherin–catenin complex may be associated with tumor development, progression, and worse prognosis [22]. In these intricate processes, it has been observed that the functional state and composition of the TME may vary considerably between patients. This is influenced by factors such as gender, age, body mass index, lifestyle, and the microbiome [23].

## 3. Cancer and the Microbiome

Apart from bacteria, a substantial number of viruses co-colonize the human gut, and are collectively referred to as the gut virome [24]. Eukaryotic and prokaryotic viruses comprise the human virome, which includes viruses that infect cells, viruses that infect microbes (including bacteria, fungi, and archaea), and viruses that originate from plants [25].

In comparison with eukaryotic viruses, human gut phages (also called bacteriophages and bacterial viruses) make up approximately 90% of the gut virome (phageome), whereas RNA phage abundance is significantly lower [24,26]. Furthermore, a large number of fungi reside in the gastrointestinal tract, collectively known as the mycobiome [27]. It has been shown that intestinal fungi are causally involved in the assembly of the microbiome and the development of the immune system [28].

Every tumor type has been found to have a different microbiome. The intratumor bacteria, which are mostly intracellular, exist in both immune cells and cancer [29]. The microbiome present within local tissues has been shown to restrict cancer development, especially by affecting the host immune system or cancer cells, but also impacting on the efficacy of cancer therapies [30]. However, some microorganisms, which may be considered infectious pathogens and potentially modifiable risk factors, can be harmful to the health of human beings. For instance, it has been estimated that 25 cases of cancer per 100,000 person-years were infection-attributable cancer cases in 2018, with *Helicobacter pylori*, hepatitis B and C viruses, and human papillomavirus (HPV) being the primary causes [31].

**Figure 1:**
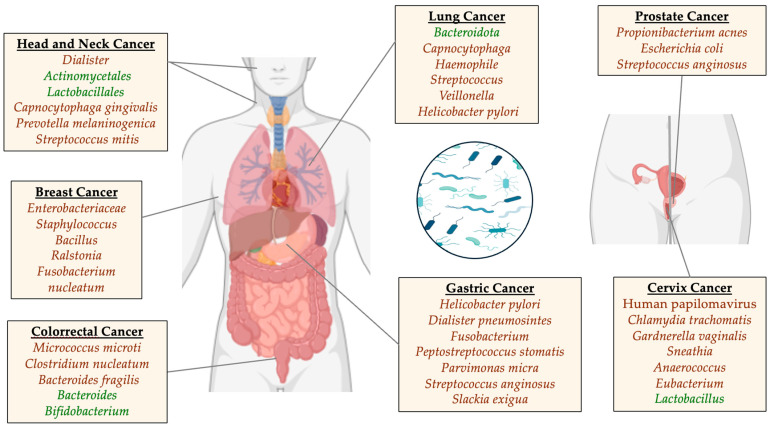
Some of the primary microorganisms related to cancer processes (in red those whose presence appears to be a risk factor and/or abundance increases during cancer; in green those whose abundance decreases during the cancer process and who apparently have a protective effect).

Intratumorally, microorganisms also affect the effectiveness of tumor chemotherapy, being a potential target in the development of novel therapies [32]. The relationship between different host-intrinsic microorganisms and a patient’s microbial composition has been postulated as a key element of future precision-medicine approaches [33]. An enhanced comprehension of the TME and its associated microbiome is still needed, remaining challenging due to the low microbial biomass in tumors [34]. Evidence regarding microbes and different types of cancer is described in the text below and graphically summarized in Figure 1.

Multiple malignancies, including colorectal, gastric, and pancreatic cancers, have been linked to pathogenic microbes harboring specific virulence factors [35]. A growing body of evidence suggests that the gut virome contributes to cancer onset and development, particularly gastrointestinal cancer. The metagenomic analysis of stool samples from patients with colorectal cancer revealed a distinct fecal virome that was richer and more diverse than the intestinal virome [36]. The growing body of evidence indicates that the gut mycobiome can strongly influence the immune system of the host and that this interaction is mediated by bacteria [37,38].

### 3.1. Carcinogenesis and Oncogenesis Processes Associated with Microbe Metabolism 

The mechanisms involved in carcinogenesis are complex and include a number of factors contributing to the transformation of malignant cells, tumor growth, and metastasis. There has been a growing interest in recent decades in the role of the symbiotic microbiome in regulating metabolism and immune function in humans [39]. The microbiome can influence the malignant transformation of the host’s cells (oncogenesis) by modulating immune system activity [40]. It may also foster mutually beneficial relationships with the host, such as barriers that prevent pathological effects. Nevertheless, these barriers can be disrupted, causing oncogenic transformations and inflammatory diseases. This occurs when microbes and the host immune system are confronted with conditions completely different from those under which they co-evolved [41]. As the tumor progresses, microorganisms produce metabolites that accumulate in the TME. These microbial metabolites act as ligands for specific receptors or cytokines to regulate protein activity and alter the TME [42]. Collectively, these changes can act as biomarkers in the context of tumor immune surveillance. For instance, various microbial markers have been investigated in relation to cancer. Key aspects of these findings will be discussed in more detail.

#### 3.1.1. Metabolic Pathway: Tryptophan-Indoleamine-Arginine-Citrulline

Metabolites from the microbiome may affect different host cell processes, including the generation of toxic products (e.g., acetaldehyde, nitrosamines, etc.), which could result in the metabolic activation of carcinogens [43]. Moreover, several major catabolic and biosynthetic pathways are interconnected with the amino acid metabolism. In the synthesis of creatine, urea, and agmatine, arginine is an important precursor [44]. An important component of the tryptophan metabolism is its microbial conversion to indole compounds, particularly in colon cancer [45]. In the TME, the microbiome plays a key role in malignant cell transformation, tumor growth, and response to treatment. During the early stages of tumor growth, changes in the tryptophan metabolism have been observed as an adaptive mechanism by which malignant cells evade immunosurveillance and spread [46].

The enzyme indoleamine 2,3-dioxygenase 1 found in *Bacillota* plays a key role in limiting the catabolism of tryptophan [47]. Species from *Clostridium*, *Ruminoclostridium*, *Lachnoclostridium*, and *Roseburia* genera express high levels of Indoleamine 2,3-dioxygenase 1 [47], and pro-inflammatory cytokines stimulate the expression of this enzyme [48]. During this pathway, indoleamine 2,3-dioxygenase 1 converts tryptophan into *N*-formylkynurenine, which is rapidly converted into kynurenine (the first stable catabolite). Kynurenine is converted into metabolites that regulate immune cell activity [48]. It has been demonstrated that kynurenine regulates immune homeostasis [49] by reducing the number of activated T cells, dendritic cells, and natural killer cells and by eliciting apoptosis of Th1 cells to prevent excessive inflammation [45].

In addition to its importance for the synthesis of kynurenine and serotonin, tryptophan also has the potential to activate the immune system and lead to the accumulation of potentially neurotoxic compounds. These characteristics make the kynurenine pathway a promising therapeutic target for treating inflammation and certain neurological diseases, particularly in cancer patients receiving chemotherapy [50]. Two major enzyme families degrade L-Arginine, namely, nitric oxide synthases and arginases. The three types of nitric oxide synthases (NOS1, NOS2, and NOS3) catalyze the conversion of L-arginine into L-citrulline, which is recycled back into L-arginine by argininosuccinate lyase and argininosuccinate synthetase [51].

#### 3.1.2. Tumor and Microbial Markers

The six types of tumor biomarkers, biologically indicating the pathogenic processes or pharmaceutical responses to therapeutic interventions, are classified into the following categories: early detection, diagnosis, prognosis, prediction, therapeutic target, and surrogate end point biomarkers [52,53]. In order to detect cancer at an early stage, biomarkers of early detection are vital. Consequently, prognostic biomarkers can be used to identify patients at a high risk of developing cancer [53,54]. In this context, there is evidence that changes in the gut microbiome may occur during the earliest stages of carcinogenesis. The presence of these changes can be used to identify patients at risk for cancer. In addition to improving screening strategies, changes in the microbiome over time may serve as biomarkers for early neoplasia detection [55]. In populations with borderline quantitative fecal immunochemical test results, microbial markers may be used as a complementary test [56]. Regarding microbiome and host interactions, the effects mediated by the immune system are of great interest. In this line, the pattern recognition receptors (PRRs) have been examined in different cancer types. These PRRs are expressed by different host cells and mediate the interaction between the microbiome, cells, and the immune system. One example of these PRRs would be the toll-like receptors (TLRs), whose expression patterns have shown to differ during cancer [43].

**Figure 2:**
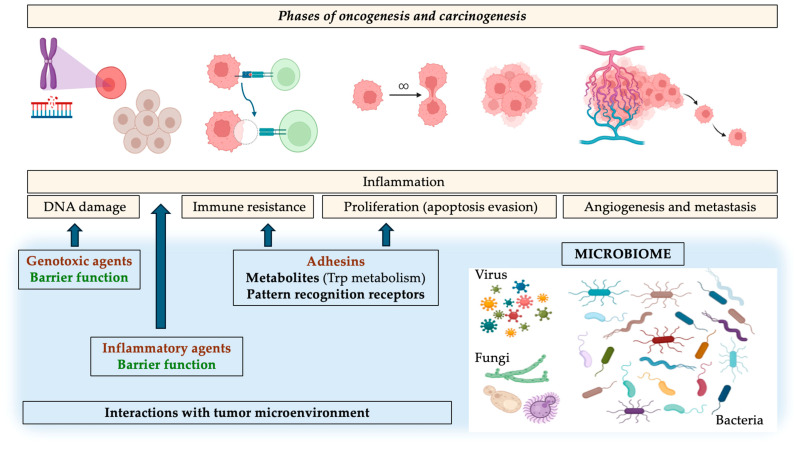
Potential role of the microbiome in different phases of oncogenesis and carcinogenesis. In red, those deleterious effects related to oncogenesis/cancer; in green, beneficial and protective effects; in black, those effects which may be deleterious or beneficial depending on the microorganism. Abbreviations: Trp, tryptophan.

Finally, this potential role of the microbiome in different phases of oncogenesis and carcinogenesis are graphically summarized in Figure 2.

### 3.2. Lung Cancer

Lung cancer leads the newly diagnosed cancer cases and the cause of cancer mortality worldwide [11]. It is often diagnosed at an advanced stage and its etiology is mostly attributable to tobacco smoking. However, epidemiological findings have shown that the rate of lung cancer in never-smokers (around 25%) is increasing [57]. Lung cancer is a heterogeneous disease comprising several subtypes which may be classified into two main groups: small-cell lung carcinoma (SCLC, the most fatal form of lung cancer) and non-small-cell lung carcinoma (NSCLC, the most common form of lung cancer). NSCLC may be further classified into squamous cell carcinoma (SCC), adenocarcinoma, and large-cell lung carcinoma [58]. 

Regarding the respiratory tract microbiome, there are substantial differences between the upper and lower respiratory tracts. In healthy individuals, the predominant bacteria in the lower airways are *Veillonella*, *Prevotella*, and *Streptococcus*, with additional bacteria including *Fusobacterium* and *Haemophilus*. The oral microbiome is the primary source of these bacterial communities [59]. Many studies have highlighted the role of the gut–lung axis in regulating the physiological activities and immune status of the lungs through different metabolic derivatives [60]. In lung cancer, *Klebsiella*, *Acidovorax*, *Polarmonas*, *Rhodoferax*, *Xylobacter*, *Eufluobacter*, and *Clostridium* are frequently found in SCLC, while *Prevotella* and *Pseudobutyrivibrio ruminis* appear to be negatively correlated with SCLC [61]. Patients affected by NSCLC have shown a higher abundance of *Ruminococcus* spp., *Akkermansia muciniphila*, *Eubacterium* spp., and *Alistipes* spp., and a lower abundance of *Bifidobacterium longum* and *adolescentis*, and *Parabacteroides distasonis* [62]. Thus, several studies have suggested that the gut microbiome may be of importance in patients suffering from lung cancer [63,64].

However, the microbiome of the lower airways appears to be also of significant relevance in homeostatic processes, with the *Bacteroidota* phylum dominating the healthy lung microbiome and gammaproteobacteria being associated with pathological processes [59]. Cancer-related microbes are also differentially abundant in lung tumor tissues. Namely, according to bacterial abundance in tumor tissues and the sputum of lung cancer patients, there would be three categories of microbes: (I) lung tumor microbes (e.g., *Capnocytophaga* and *Haemophile*), (II) sputum microbes of lung cancer patients (e.g., *Veillonella*, *Streptococcus*, and *H. pylori*), and (III) bacterial genera whose changes are consistent with those in tumor tissues (e.g., *Acidovorax* and *Capnocytophaga*) [65]. The presence of all these microorganisms may adversely affect both cancer treatment and the cancer process, which seem to be linked to pulmonary dysbiosis [66].

### 3.3. Head and Neck Cancer

Head and neck cancer (HNC) accounts for approximately 7.6% of all cancer cases worldwide [67]. It predominantly affects men and individuals over the age of 50. Major risk factors include tobacco and alcohol consumption, HPV and Epstein–Barr virus infections, and exposure to certain chemicals and radiation [68]. Most HNCs are SCCs (HNSCCs) arising from the mucosal epithelium of the oral cavity, nasopharynx, oropharynx, hypopharynx, and larynx [69].

An emerging area of research is the impact of the microbiome on the TME of HNSCC, with a particular focus on oral cancer [69]. Poor oral/dental health, in conjunction with tobacco use and alcohol consumption, is closely linked to oral cancer [70] and, additionally, these factors could modify the oral microbiome [71]. A comparison of the oral microbiome between patients with HNC and controls revealed significant differences in microbial composition and function [72]. For instance, cancer cases exhibited increased levels of *Dialister* and decreased abundance of *Actinomycetales* and *Lactobacillales*. Functional analysis showed higher levels of genes involved in lipopolysaccharide (LPS) synthesis and reduced LPS transport, indicating a shift towards Gram-negative bacteria. Changes in amino acid metabolism, including increased serine synthesis and decreased glutamate uptake, suggest a transition to anaerobic metabolism [73]. Additionally, HNC cases showed altered synthesis and transport of vitamins and metals, reflecting an adaptation to a less oxidative environment and changes in saliva osmolarity. 

In contrast, higher levels of *Corynebacterium* and *Kingella* in the oral microbiome are linked to a reduced risk of HNSCC, possibly due to their role in carcinogen metabolism [74]. This association is especially notable in larynx cancer and among tobacco users.

Specific viral, bacterial, and fungal signatures that are enriched in oral cancer have been identified and are continuously being refined [69]. The use of pan-pathogen array technology has led to the identification of microbiome signatures specific to oral cancer [75]. Distinct microbial patterns associated with oral cancer have been revealed, including HPV16 viral signatures and bacterial taxa such as *Brevundimonas*, *Escherichia*, *Alcaligenes*, *Comamonas*, *Caulobacter*, *Cardiobacterium*, *Serratia*, *Plesiomonas*, *Haemophilus*, *Edwardsiella*, *Frateuria*, *Rothia*, and *Peptoniphilus*. Additionally, fungal signatures like *Geotrichum*, *Rhodotorula*, and *Pneumocystis*, as well as parasitic signatures such as *Centrocestus*, *Hymenolepis*, and *Trichinella*, are uniquely associated with oral cancer. These findings suggest that these microorganisms may play roles in initiating, promoting, or modulating cancer or may grow in the TME. The oral cancer-specific microbiome signatures hold potential as biomarkers for diagnosis and prognosis. Moreover, a microbe–host fusion map spanning human chromosomes has been developed, which may provide insights into how microbial interactions influence host gene expression and contribute to oral cancer development [75].

For these reasons, the salivary microbiome has emerged as a potential diagnostic indicator of oral cancer. In a comparative analysis, salivary counts of 40 common oral bacteria in subjects with an oral SCC lesion differed from those found in cancer-free controls [76]. Notably, higher levels of *P. melaninogenica*, *C. gingivalis*, and *S. mitis* were found to be associated with oral cancer. These elevated bacterial levels suggest that the presence of these microorganisms in saliva could serve as biomarkers for the early detection and diagnosis of oral cancer. This is particularly significant given that approximately half of oral cancers are diagnosed at advanced stages (III–IV) [77], which impacts prognosis and survival [78]. Therefore, enhanced screening tools are needed, and salivary biomarkers could serve as an easy and non-invasive tool.

### 3.4. Breast Cancer

Women worldwide suffer from breast cancer at a rate that ranks second among all cancer-related deaths [79,80]. Malignancies of this type are heterogeneous, and distinctive molecular subtypes have been identified [81]. There is a subgroup called luminal A, which is described by the activity and expression of estrogen receptors (ERs) and has the best clinical outcome due to its good reaction to endocrine therapy. In contrast to the previous subtype, luminal B cancers have minor levels of ERs and have a greater rate of proliferation [82]. A subgroup of invasive breast cancer that is positive for human epidermal growth factor receptor 2 is ER- and progesterone receptor (PR)-negative, and represents about 15% of all invasive breast cancers. Tumors of this type are more aggressive than tumors of the luminal type [81]. As a final point, triple-negative breast cancers, the subtype with the lowest survival and that is the most difficult to treat, do not express hormone receptors or human epidermal growth factor receptor 2 [81].

Breast cancer is consistently linked to breast-tissue-resident bacteria [83], but their origin remains unclear, and various hypotheses have been advanced. Breast cancer patients presented higher levels of *Enterobacteriaceae*, *Staphylococcus*, and *Bacillus* compared to healthy individuals, according to Urbaniak et al. [83]. Among the bacterial genera found in mammary tissue, Costantini et al. [84] identified *Ralstonia* as the most abundant. *Fusobacterium nucleatum*, a pathogen of the oral cavity, was found to translocate via the bloodstream and accumulate in breast tumors [84]. In a study published in The Cancer Genome Atlas, Thompson et al. characterized the microbiome of breast tumor tissues and non-cancerous adjacent tissues. Based on their findings, it appears that microbial composition has changed among the disease subtypes. *Pseudomonadota* were more abundant in tumor tissues, while *Actinobacteriota* were more abundant in non-cancerous adjacent tissues. Additionally, they found an association between *Listeria* spp. and genes involved in epithelial-mesenchymal transitions [85].

Breast cancer is closely associated with the microbiome of the gut [86]. Through a variety of mechanisms, intestinal dysbiosis can favor certain microorganisms that inhabit our intestines to increase our risk of developing certain types of breast cancer [87]. 

### 3.5. Gastric Cancer

Gastric cancer, or stomach cancer, is a condition characterized by the formation of malignant cells within the gastric lining. This disease typically evolves over several years and may originate in various regions of the stomach [88]. According to Global Cancer Statistics 2020, gastric cancer accounted for approximately 1.089 million new cancer cases and 0.7 million deaths globally in 2020. This makes it the fifth most prevalent malignancy and the fourth leading cause of cancer-related mortality [89]. The incidence of gastric cancer exhibits significant geographical variability, with the highest prevalence observed in Eastern Asia. It also exhibits a marked gender disparity, as men are twice as likely to be affected compared to women. Notably, a regression in the gastric cancer incidence has been observed in North America and Western Europe over the past decade [90]. 

Despite the significant role of environmental factors, lifestyle, and hereditary characteristics in gastric cancer, dietary habits and microbiome characteristics are the primary contributors [91]. Gut microbiome dysbiosis has been implicated in tumorigenesis, with gastric cancer serving as a prototypical example of the interaction between gut microbiome dysbiosis and the host’s epithelial cells [92]. *Helicobacter pylori* infection is the most well-documented risk factor for non-cardia gastric cancer, and is estimated to be responsible for 65% to 80% of all gastric cancer cases [93]. Chronic *H. pylori* infection leads to a stepwise progression from atrophic gastritis to intestinal metaplasia [94]. The oncogenic effects of *H. pylori* are mediated through two primary mechanisms: an indirect inflammatory response within the gastric mucosa and a direct epigenetic impact on gastric epithelial cells [95]. Besides *H. pylori*, a predominance of *Lactobacillus* spp., *Veillonella* spp., and *Clostridium* spp. has been observed, as these bacteria are resistant to the acidic gastric environment [96]. 

Other species, such as *Escherichia coli*, *Peptococcus* spp., *Klebsiella* spp., and *Bacteroides* spp., have also been identified but remain dormant due to the stomach’s acidity. Sequencing-based studies of the gastric microbiome consistently reveal that *H. pylori* dominates the gastric niche when present, thereby shaping the composition of the bacterial communities within the stomach [97].

Oral bacteria, including *Fusobacterium*, *Dialister pneumosintes*, *Peptostreptococcus stomatis*, *Parvimonas micra*, *Streptococcus anginosus*, and *Slackia exigua*, have been observed in higher abundance in patients with gastric cancer [98]. Additionally, microbial gut dysbiosis has been linked to gastric carcinogenesis. This dysbiosis is characterized by a reduction in *Neisseria*, the TM7 group, *Porphyromonas*, *Streptococcus sinensis*, and *Prevotella pallens*, and coupled with an enrichment of *Klebsiella pneumoniae*, *Lactobacillus coleohominis*, *Lachnospiraceae*, and *Acinetobacter baumannii* [99].

### 3.6. Colorectal Cancer

Colorectal cancer, a malignancy originating in the colon or rectum, is commonly designated colon cancer or rectal cancer depending on its initial site [100]. Colorectal cancer ranks among the most prevalent malignant tumors globally. As of 2020, the incidence and mortality rates have ascended to the third and second highest worldwide, respectively [101]. It is the second most frequently diagnosed cancer in women and the third in men, with mortality and incidence rates approximately 25% lower in women compared to men. Additionally, these rates exhibit geographical variability, with the highest incidences observed in more developed countries [102].

Currently, it is widely acknowledged that, in addition to genetic factors, inflammatory bowel disease mediated by gut microbiome dysregulation is a primary causative factor in the development of colorectal cancer. Patients with colorectal cancer frequently exhibit an increased presence of pro-inflammatory bacterial species, including *Clostridium nucleatum*, *Micrococcus microti*, and *Bacteroides fragilis*, in comparison with samples from control patients. In contrast, these samples from control patients generally display a greater abundance of favorable bacteria such as *Bifidobacterium* and *Bacteroides* species [103]. This disparity in bacterial profiles highlights the potential role of specific bacterial species in either inhibiting or promoting colorectal cancer development [104]. Through a variety of mechanisms, the gut microbiome influences colorectal carcinogenesis, including immune response regulation, inflammation, and altered dietary metabolism, as well as the production of harmful microbial-derived products, including genotoxins and metabolites [105,106,107,108,109,110,111].

Among the most significant bacteria linked to colorectal cancer, called colorectal cancer-associated bacteria, are *Enterococcus faecalis*, *Bacteroides fragilis*, *Streptococcus gallolyticus*, and *Escherichia coli* [112]. The presence of increased numbers of *Prevotella*, *Parvimonas*, *Fusobacterium nucleatum*, *Porphyromonas*, and *Peptostreptococcus* in fecal and tumor samples from patients with colorectal cancer has been identified [105,106]. These bacteria are considered potential pathobionts, defined as resident microbes with pathogenic potential. Although pathobionts may be innocuous to the host under normal conditions, many exhibit high abundance in colorectal cancer and are related to chronic inflammatory conditions [113]. Interestingly, in colorectal cancer, there is higher knowledge regarding the specific role of the microbes in the cancer process. In this line, three microbes have been associated repeatedly with cancer development: (I) *Escherichia coli*, which would be responsible for cancer initiation producing colibactin (a genotoxic molecule that induces DNA damage), (II) *Bacteroides fragilis* promoting inflammation and toxin-induced cell proliferation, and (III) *Fusobacterium nucleatum* producing Fap2 and FadA (adhesins that contribute to proliferation, immune evasion, and, possibly, metastases) [114].

### 3.7. Prostate Cancer

Prostate cancer is a malignancy originating from genomic alterations in the prostate, which subsequently leads to tumorigenesis [115]. Globally, it is the second most common cancer type in men, with nearly 1.4 million newly diagnosed cases and 400,000 prostate cancer-associated deaths reported in the same period [116]. In Europe, the tendency is similar, and it is also the most prevalent cancer and the third leading cause of cancer-related mortality in men, accounting for 23.2% of all newly diagnosed cancer cases in 2020 [117]. 

Recent studies suggest that alterations in microbiome composition (dysbiosis) might have a critical function in the occurrence, development, and prognosis of prostate cancer [118]. Analysis of human fecal samples indicated that, although the overall gut microbiome diversity did not significantly differ between prostate cancer patients and healthy controls, specific bacterial species were associated with the presence of prostate cancer [119]. Prostate cancer cells are influenced by direct contact with the microbiome of local tissues and urine [120]. In this context, interactions between various microbiomes and the prostate, potentially related to prostate cancer, can largely be categorized into two distinct pathways: indirect and direct. Indirect pathways comprise the gastrointestinal tract, including the oral and fecal microbiome, while direct pathways involve the prostate and urine microbiomes [121]. Among the commonly observed bacteria in the urinary microbiome, *Propionibacterium acnes* has been particularly noted in patients with prostate cancer. Additionally, pro-inflammatory bacteria such as *Escherichia coli, Streptococcus anginosus*, and *Propionibacterium acnes* have been implicated in both acute and chronic prostatitis, which may lead to hyperplasia and an increased risk of prostate cancer development [122]. It is probable that microorganisms are present in the prostate only during a pathological state. In certain instances, such as bacterial prostatitis, these microbes may be the causal agents of the disease. Alternatively, in conditions like prostatic atrophy, altered prostatic architecture may result in the breakdown of the epithelial barrier, subsequently allowing microbial infiltration [123]. Although the prostate is not precisely modified by the gut microbiome, it may be indirectly influenced by immune cells and cytokines altered by the gut microbiome, or by compounds absorbed from the intestine that enter the systemic circulation and bacterial metabolites. This phenomenon can be conceptualized as a “microbiome-gut-prostate axis” [124].

### 3.8. Cervix Cancer

Cervical or cervix cancer is the fourth most common cancer and cause of cancer-related deaths among women worldwide. Globally, one in seventy women will develop cervical cancer, although there are significant differences in incidence and mortality rates among countries [125]. It is possibly the most curable human cancer when it is detected at the precancerous stage, and millions of malignancies could be prevented through immunization and screening [126]. The major cervical cancer risk factor is the persistent infection with high-risk HPV types. HPV infection and its further progress to cervix carcinogenesis has been associated with different factors, such as long-term use of oral contraceptive pills, tobacco consumption, diet, coinfection with other sexually transmitted agents, or immunosuppression [125]. HPV and *Chlamydia trachomatis* are the most common pathogens in sexually transmitted infections, both increasing the risk of infertility and cervical cancer, and usually persisting through adult life asymptomatically and silently. Microbiome and immune status strongly influence coinfection with these pathogens [127]. Although most of the cervical HPV infections resolve spontaneously, a minority of cases persist and progress to dysplasia and cancer. In recent years, the role of the vaginal microbiome in this process has been investigated. Bacterial vaginosis, primarily caused by *Gardnerella vaginalis*, is considered an indirect marker of sexual behavior leading to HPV infection [128].

The uterine cervix is characterized by a particular acidic microenvironment created by epithelial cells and symbiotic bacteria, mainly *Lactobacillus* spp., the predominant microorganisms in the cervix and vagina. Differences have been reported in the vaginal microbiome of patients with cervical cancer, especially regarding the abundance of *Mycoplasma genitalium*, aerobic lactobacilli, *Staphylococcus epidermidis*, enterococci, *Escherichia coli*, and *Bacteroides* species [129]. Furthermore, HPV infection appears to affect the local microbiome, with decreased abundance of *Lactobacillus* spp. accompanied with higher microbial diversity. *Fusobacteria*, such as *Sneathia* spp., may serve as a potential marker of HPV infection. Additionally, an increase in *Bacteroidota* and a decrease in *Actinobacteriota* have been observed in these patients [130]. Results based on 16S rRNA and gene expression suggest that *Prevotellaceae*, *Tissierellaceae*, and *Fusobacteriaceae* are the most abundant microbes in cervical carcinoma, while other bacteria (e.g., *Anaerococcus*, *Hydrogenophilaceae*, *Eubacterium*) may be risk factors implicated in different processes such as the regulation of viral response or epithelial cell differentiation in cervical cancer [131].

## 4. Diagnostic Methods, Novel Treatments, and Future Perspectives in the Study of the Human Microbiome and Cancer

The interplay between the human microbiome and cancer is an emerging field of study. It has revealed significant insights into how microorganisms influence cancer development. These insights can have a significant role in the diagnosis and treatment of different diseases. Recent advancements in “omics” technologies and methodologies, such as metagenomics, metabolomics, metatranscriptomics, culturomics, and proteomics, have enabled researchers to delve deeper into these complex relationships, leading to novel diagnostic methods, therapeutic strategies, and future perspectives that have the potential to transform and enhance clinical oncology.

### 4.1. Cancer Diagnostic Methods through the Human Microbiome

Early cancer diagnosis is a critical first step in increasing survival rates, minimizing treatment severity, and enhancing the cancer patients’ quality of life. Early detection is key to effective treatment, but several challenges remain currently: (I) building a better comprehension of the biology of early cancer, (II) determining the risk of developing cancer, (III) searching for and confirming cancer detection biomarkers, (IV) increasing the availability of accurate technologies for early detection, and (V) assessing approaches focused on early detection [132]. These challenges may be addressed by deciphering comprehensive microbiome research (microbial signatures, functionality, metabolite production, microorganism metabolism, and/or host interaction). This can impact cancer progression in various manners: contact-dependent effects (the most well-known occur locally at the mucosal surface or within the TME), contact-independent effects (mediated by microbial metabolites and outer membrane vesicles in circulation), and immunologically mediated processes [133]. Together, these factors drive the complex host–microbe interactions that lead to microbiome-induced cancer modulation. Due to the variability across different cancer types and between malignant and healthy tissues, as described in this report, this can result in the identification of unique signatures or biomarkers that could be used for developing early diagnostic tools or even for monitoring disease stage and progression. In this line, numerous studies have highlighted the significant potential of the microbiome as a diagnostic tool for cancer detection in both observational and experimental contexts [134,135,136,137,138,139,140,141,142,143,144,145,146].

Feces, saliva, and plasma samples are the most common samples used for these analyses, which makes this methodology minimal or non-invasive for cancer detection. This characteristic enhances its usefulness as a diagnostic tool. This is especially true when it comes to the ease of obtaining these samples. In addition, the current detection techniques, such as biopsies or endoscopies, can be invasive and uncomfortable for patients. Most of these studies focus on detecting microbial signatures or specific microorganisms as biomarkers associated with cancer, with colorectal cancer being the most prominently studied [136,137,139,140,144,145]. Gastric [141], pancreatic [134], lung [135,138], and cervical cancers [143,146], among others, have also been explored. While the fecal microbiome is the first choice for identifying diagnostic biomarkers for colorectal cancer, saliva samples have also been associated with a wide range of cancers such as oral, pancreatic, lung, and even colorectal cancer. Detecting cancer outside the aerodigestive tract, such as breast, ovarian, prostate, or brain cancers, is possible using plasma, a more recent method with increasing interest. This recent procedure is based on cell-free DNA, also called liquid biopsies, and focuses on using circulating tumor DNA present in plasma [30].

Various studies have investigated the association between the gut and oral microbiome and different cancers, producing models with high sensitivity and specificity, which could be related with the progression or development of the cancer. For example, a combination of *Lachnoclostrium* spp., *Fusobacterium nucleatum*, *Hungatella hathewayi*, and *Bacteroides clarus* in the fecal microbiome showed sensitivity and specificity of 94% and 81%, respectively, for detection of colorectal cancer [139]. Similar findings and more detailed examples are discussed in a recent review by Kandalai et al. [30]. In addition to microorganisms, circulating microbial-related metabolites have also emerged as a promising cancer diagnostic tool [147,148,149,150]. In a recent study, Irajizad et al. analyzed the metabolomic profiles of 172 serum samples from patients with pancreatic cancer and compared them to 863 samples from individuals with different types of cancer. They developed a three-marker panel of microbial-related metabolites (indoleacrylic acid, trimethylamine N-oxide, and indole-derivative_2) to assess the five-year risk of pancreatic cancer. This predictive model was further enhanced by combining non-microbial metabolites and the CA19-9 marker, providing a robust tool to identify individuals at high risk of pancreatic cancer who could benefit from enhanced surveillance and cancer interception strategies [148]. In another multi-omics study, distinct microbiome–metabolome associations were observed at different colorectal cancer stages [149]. A depletion of short chain fatty acids and reduced gamma-aminobutyric acid biosynthesis were reported in early-onset colorectal cancer, while an increase in tryptophan, bile acid, and choline metabolism were detected in late-onset colorectal cancer. These findings offer a promising approach for the detection and identification of patients with this disease [149].

However, the issue of the replicability of these biomarkers across different cohorts and populations arises due to unique microbiome specificity. A meta-analysis of 969 fecal metagenomes revealed consistently high accuracy in predictive microbiome signatures for colorectal cancer across multiple datasets, which enhances the credibility of reproducible microbiome biomarkers and accurate disease-predictive models that can support clinical cancer prognostic tests [151]. Conversely, a recent study utilizing quantitative microbiome profiling based on 16S ribosomal RNA amplicon sequencing, combined with rigorous confounder control, found that the well-established colorectal cancer microbiome markers, such as *Fusobacterium nucleatum*, did not significantly associate with colorectal cancer groups, while other associations (*Dialister pneumosintes*, *Anaerococcus vaginalis*, *Parvimonas micra*, *Porphyromonas asaccharolytica*, *Peptostreptococcus anaerobius*, and *Prevotella intermedia*) remained robust [145]. The complexity of these studies necessitates rigorous and well-described methodologies to ensure reproducibility and advance the field. Recently, significant and promising findings regarding the discovery of unique microbial signatures in tissue and blood across over 30 types of cancer suggested the potential for microbiome-based oncology diagnostics using solely plasma-derived, i.e., cell-free, microbial nucleic acids [152]. However, major errors in data analysis resulted in false-positive findings and artificial signatures, invalidating the results and leading to the recent retraction of the article [152]. This underscores the importance of accounting for recognized confounders and variables that affect results. It highlights that, while cancer microbiome research is promising, continued refinement is necessary. The accuracy, consistency, and prevalence of these biomarkers during different cancer treatments, as well as their behavior in conjunction with adjunctive therapies like antibiotics, comprise another challenging step that must be resolved before clinical implementation. Although these techniques are promising for early-stage cancer detection and reducing the necessity for invasive diagnostic procedures, microbiome-based diagnostics are likely to complement existing methods such as biopsies and imaging, rather than serve as standalone approaches in the near future. Although microbiome-based diagnostics have the potential to evolve into independent and primary diagnostic tools, we are still in the early stages. Much remains to be elucidated and understood about the complex spatial and functional interactions between microbiomes and cancer.

### 4.2. Novel Cancer Treatments through the Microbiome

Cancer treatment typically includes a combination of surgery, chemotherapy, and radiation. Although many advances have been made in this area, it is necessary to improve treatment efficiency for specific types of cancer or cases with low response rates or resistance to chemotherapy and radiotherapy [153]. There is evidence that the microbiome has an important function in tumorigenesis, with dense colonies of bacteria inhabiting both cancerous and immune cells [29]. These microorganisms interact with the host through complex pathways, shaping the immune landscape and modifying cytokine profiles and local immune activities. These interactions can promote or suppress cancer, underscoring their potential as crucial elements in cancer therapy. Additionally, the exogenous application of engineered microorganisms to mediate cancer immunotherapy, along with probiotics or fecal microbiome transplantation, represents other promising avenues in the interaction between the microbiome and cancer therapy. This emphasizes the need for further exploration of the microbiome’s role in developing more effective cancer treatments.

Certain intratumoral bacteria are linked to chemotherapy resistance in cancer due to endogenous enzymes that metabolize chemotherapeutic drugs, altering their effectiveness and toxicity [154]. For instance, the long isoform of the bacterial enzyme cytidine deaminase found in *Gammaproteobacteria* can modify the biological structure of the chemotherapy drug gemcitabine, which makes it ineffective and contributes to chemoresistance in pancreatic ductal adenocarcinoma, which could be countered with the use of the antibiotic ciprofloxacin [155]. Similarly, *Fusobacterium nucleatum* was abundantly found in colorectal cancer tissues of patients with post-chemotherapy recurrence. This promotes resistance to 5-fluorouracil and oxaliplatin by targeting toll-like receptor-4 and MYD88 innate immune signaling and specific microRNAs (miR-18a* and miR-4802). Thus, it activates the autophagy pathway and alters the cancer’s response to chemotherapy [156]. On the other hand, the microbiome can also alter the toxicity profile of these drugs. *Akkermansia muciniphila* and *Lactiplantibacillus plantarum* have demonstrated the ability to reduce mucositis side effects induced by 5-fluorouracil. This action may be produced throughout mechanisms such as restoring intestinal barrier function and suppressing eosinophil peroxidase and myeloperoxidase activity [157,158]. Additionally, bacterial β-glucuronidases can convert the inactive form of the bio-transformed irinotecan drug into its active form, which is toxic and causes treatment-limiting diarrhea [159]. Targeting specific bacterial species is not the most effective approach to preventing this, given the widespread presence of these enzymes. Instead, combining this drug with glucuronidase inhibitors has been proposed to prevent diarrhea. However, this approach requires selectivity since host-derived β-glucuronidases can also transform the drug [159]. The microbiome’s dual role in counteracting and enhancing chemotherapy effectiveness highlights the importance of further study. Exploring these interactions in full depth could lead to adjuvant treatments that optimize therapeutic outcomes. This is due to the specific roles of both drugs and microbiome. 

Radiotherapy is another alternative cancer treatment, and the microbiome has also been implicated in its side effects. This is mainly focusing on the gut microbiome due to radiation enteropathy, which resembles the pathophysiology of inflammatory bowel diseases. Preclinical and clinical data reliably support that component of the gut microbiome, (e.g., short chain fatty acids, lactate) and *Akkermansia* provides intestinal and hematopoietic radioprotection [160]. It has been reported that vancomycin-sensitive bacteria, which target mostly Gram-positive bacteria, enhance the antitumor activity of radiotherapy in preclinical models of melanoma, lung and cervical tumors. This enhancement is dependent on the cross presentation of tumor-associated antigens to cytolytic CD8+ T cells and on IFN-γ production [161]. Shiao et al. showed that gut fungi modulate antitumor immune responses following radiation in mouse models of melanoma and breast cancer, with bacteria and fungi exerting opposing effects on these responses [162]. The fungi depletion through gnotobiotic methods enhanced radiation responsiveness, whereas antibiotic-mediated depletion of bacteria reduced this responsiveness and led to an overgrowth of commensal fungi. Additionally, higher intratumoral Dectin-1 expression, a key innate sensor of fungi, was correlated with poorer survival in patients with breast cancer. It was essential for the commensal fungi effects in radiation therapy mouse models [162]. Although less research has been conducted on the role of the microbiome in response to radiotherapy compared to other cancer treatments, existing studies indicate that the microbiome may significantly influence patient responsiveness to this therapy.

Immunotherapy has demonstrated promising effects in recent years, offering new approaches to cancer treatment. These advances are due to our improved immune system comprehension and its mechanisms. These understandings are necessary for effectively leveraging the body’s natural defenses to combat cancer. Since tumor cells often possess unique tumor antigens, immunotherapy works by binding to these antigens and activating the immune system to recognize and kill cancer cells. An alternative strategy in immunotherapy is to inhibit immune checkpoints, such as cytotoxic T-lymphocyte-associated antigen 4 (CTLA-4) and programmed cell death protein 1 (PD-1), which typically suppress T cell function. The gut microbiome modulates response to anti-PD-1 immunotherapy in patients with melanoma [163]. Patients with a high abundance and diversity of the *Ruminococcaceae* family exhibited improved systemic and antitumor immune responses compared to patients with low diversity and high relative abundance of *Bacteroidales*, suggesting that a healthy gut microbiome could help patients combat cancer [163]. A recent study identified down-regulation of the pathway of PD-L2-RGMb as an exclusive mechanism by which the gut microbiome can stimulate responses to PD-1 checkpoint blockade in melanoma patients [164]. Additionally, anti-CTLA-4 immunotherapy efficacy depends on gut microbiome-immune interactions and has been shown to be most effective when the gut microbiome is present. After antibiotic treatment, recolonization with *Bacteroides fragilis*, *Bacteroides thetaiotaomicron*, *Burkholderia cepacia*, or a combination of these bacteria improved the efficacy of anti-CTLA-4 treatment by mediating anti-CTLA-4 responses through interleukin-12-dependent Th1 immune signaling in mice [165]. Also, increased abundance of *Faecalibacterium prausnitzii* and *Gemmiger formicilis* has been associated with an enhanced response to anti-CTLA-4 in patients with metastatic melanoma [166]. The results also define a potentially effective immunological strategy for treating patients who do not respond to PD-1 cancer immunotherapy. 

Recent advancements have highlighted the potential of engineered microorganisms as a strategy in cancer immunotherapy [167,168]. By leveraging synthetic biology and genetic engineering, both bacteria and viruses can be designed to specifically target cancer cells and modulate the immune system. Genetically modified bacteria can be programmed to express genes or regulatory elements that boost therapeutic effects. An example of this is *Listeria monocytogenes* engineered to produce tumor-associated antigens, which activate the immune system to identify and attack cancer cells, or *Escherichia coli* that has been engineered to secrete immunomodulatory proteins such as cytokines or checkpoint inhibitors, enhancing the immune response against tumors [168]. Moreover, bacteria can be tailored to locate hypoxic tumor regions, where they release cytotoxic agents to eliminate cancer cells and attract immune cells. On the other hand, oncolytic virotherapy employs natural or genetically altered viruses to selectively infect and destroy tumor cells, minimizing damage to normal cells. These oncolytic viruses also have the capability to stimulate the immune system to mount a robust attack against tumors [168]. These innovative techniques showcase the promising role of engineered microorganisms in improving cancer immunotherapy outcomes.

### 4.3. Outlook and Future Perspectives in the Study of the Human Microbiome and Cancer

The future of microbiome research in cancer holds immense promise for both therapeutic and diagnostic applications. A major challenge is individual microbiome variability, which complicates predicting patient responses to cancer treatments. Interindividual differences in microbiome composition and function necessitate a personalized approach that aligns with precision medicine. External factors such as antibiotics, prebiotics, probiotics, and patient’s lifestyle can disrupt the microbiome, affecting the efficacy of cancer therapies. In some contexts, these factors can help manage microbial populations to enhance treatment effectiveness. Balancing these disruptive and supportive roles is crucial for optimizing cancer outcomes, requiring careful consideration in designing and interpreting clinical trials. Promising research avenues include adjusting treatments to specific microbiome profiles to enhance therapeutic efficacy and minimize adverse effects. This approach demands comprehensive profiling of patients’ microbiomes and understanding how different microbial communities interact with cancer treatments. Achieving these goals requires more multi-omics data, stronger causality evidence, and clearer mechanisms to facilitate customized therapeutic strategies.

We must advance further and gather more information to corroborate the results obtained. To achieve this, large-scale, multi-center studies are essential to standardize methodologies for sample collection and data analysis, ensuring reproducibility and comparability across investigations. Longitudinal studies tracking microbiome changes during cancer treatment can identify microbial signatures associated with treatment response or resistance, paving the way for personalized microbiome-based therapies, and integrating genomics, transcriptomics, and metabolomics to provide deeper insights into the complex interactions between the microbiome, cancer cells, and immune system.

Recognizing confounders and variables that affect results underscores the need for continued refinement in cancer microbiome research. Addressing the accuracy, consistency, and prevalence of biomarkers, as well as their interaction with adjunctive therapies like antibiotics, is crucial before clinical implementation. In conclusion, while microbiome and cancer research is still evolving, the potential for innovative diagnostic and therapeutic strategies is immense. Addressing interindividual variability, antibiotic dual roles, and leveraging personalized and engineered microbiome therapies can transform cancer care. Continued exploration and collaboration promise improving patient outcomes and new hope in the cancer fight. Understanding the mechanisms by which the microbiome modulates treatment response and cancer progression is essential for advancing this research and developing more effective targeting strategies. As this field evolves, it represents a significant advancement in medical oncology by offering new prospects for more personalized and effective treatments.

## 5. Conclusions

The tumor microbiome is organ-specific and there are several relationships between microbes and cancer. Each type of cancer alters the composition of the tissue microbiome and the gut microbiome. It is possible that these processes contribute to the progression or development of cancer. Developing models with high sensitivity and specificity and developing new and personalized therapies could be achieved by identifying the underlying mechanisms, interactions, and biomarkers. It is expected that continued exploration and collaboration will improve patient outcomes and provide new hope for the cancer fight. As this field develops, more personalized and effective treatments are possible, representing a significant advancement in medical oncology.

## Figures and Tables

**Table 1:** Global cancer incidence in 2022: the 10 major types. Estimations from IARC [11].

Cancer Type	Number of Cases	ASR	Crude Rate	Cumulative Risk 74
Trachea, bronchus and lung	2,480,675	23.6	31.5	2.9
Breast	2,296,840	46.8	58.7	5.1
Colorectum	1,926,425	18.4	24.4	2.1
Prostate	1,467,854	29.4	37.0	3.7
Stomach	968,784	9.2	12.3	1.1
Liver and intrahepatic bile ducts	866,136	8.6	11.0	1.0
Thyroid	821,214	9.1	10.4	0.91
Cervix uteri	662,301	14.1	16.9	1.5
Bladder	614,298	5.6	7.8	0.64
Non-Hodgkin lymphoma	553,389	5.6	7.0	0.60
All cancers excl. non-melanoma skin cancer	18,741,966	186.5	237.7	19.2

Abbreviations: ASR = age-standardized rate; IARC = International Agency for Research on Cancer. Specifications: Data include both sexes, and cover 185 countries and 36 cancer types. ASR represents a summary measure of the rate if the population had a standard age structure. The crude rate for a specific tumor is calculated by dividing the number of newly occurring cancer cases observed during a given period by the corresponding number of individuals; the results are expressed as an annual rate per 100,000 individuals at risk. Cumulative risk is the probability of developing the disease during a specified period. It is expressed as the number of newborn children (out of 100) who are expected to develop a particular cancer over a lifetime (commonly defined in the age range 0–74 years). The table does not include head and neck cancer as it does not rank among the top 10.
